# Effects of High-Energy Extracorporeal Shockwave Therapy on Pain, Functional Disability, Quality of Life, and Ultrasonographic Changes in Patients with Calcified Rotator Cuff Tendinopathy

**DOI:** 10.1155/2022/1230857

**Published:** 2022-03-04

**Authors:** Arooj Fatima, Ashfaq Ahmad, Syed Amir Gilani, Haider Darain, Shiza Kazmi, Kamran Hanif

**Affiliations:** ^1^University of Lahore, University Institute of Physical Therapy, Lahore, Pakistan; ^2^University of Management & Technology, Lahore, Pakistan

## Abstract

**Objective:**

The current trial was designed to evaluate the effects of high-energy shockwave therapy on objective and subjective outcomes among participants with calcified rotator cuff tendinopathy.

**Methods:**

This parallel-group, randomized trial consists of 42 patients affected by calcific tendinopathies divided into two groups of 21 participants. Patients having calcified tendinopathy aged between 30 and 65 years with type A or B calcification were selected in the trial after signing the written consent form. Participants in the ESWT+RPT group received eight sessions of shockwaves, while the RPT group was treated by routine physical therapy. About 2000 shockwaves of 0.32 mJ/mm^2^, 120 Hz per treatment, were given as 12 sessions for the first six weeks (2 sessions/week). Pain intensity and shoulder functional ability, ultrasonographic changes, and quality of life were assessed with the numeric pain rating scale (NPRS), Constant-Murley score (CMS), ultrasonography, and Western Ontario rotator cuff index (WORC).

**Results:**

There were significant differences regarding NPRS and CMS between the two groups, at baseline and 6th and 12th weeks after intervention (*p* < 0.05). Within-group differences also showed statistically significant results after treatment (all *p* < 0.05). Significant results were seen in the WORC and ultrasonographic results pre- and posttreatment; more significant findings were found in the experimental group as compared to others.

**Conclusion:**

High-energy shockwave therapy has been proved to be effective and thus strongly recommended for the management of calcified rotator cuff tendinopathy, improving the pain, functionality, and quality of life of these participants and decreasing the size of calcified deposits. Shockwave therapy is proved to be superior to routine physiotherapy.

## 1. Introduction

One of the most prevalent musculoskeletal disorders is shoulder pain; almost 6.9 to 26 percent of adults experience this pain in their lifetime [1]. A common cause for the painful shoulder is rotator cuff (RC) injury, and among the most vulnerable tendons is the supraspinatus tendon (ST) affecting the quality of life of the patient. Because of its location in the subacromial space, ST is considered more vulnerable [2]. In fewer cases, it can be relatively asymptomatic, and proper diagnostic imaging can be required [3]. In RC calcific tendinopathy, the deposition of hydroxyapatite crystals occurs in RC tendons, most commonly the ST and infraspinatus tendons. The prevalence of RC calcific deposits in asymptomatic adults is 7.8 percent, and with the presence of subacromial pain syndrome (SAPS), the prevalence is about 42.5 percent [4].

Due to multifactorial etiology, RC tendinopathy involves both extrinsic and intrinsic mechanisms [5]. Extrinsic factors include anatomical anomalies of the acromion, deficient RC and scapular muscle performance, postural faults, kinematics of humerus and scapula changes, and reduced stretchability of pectoralis minor that affect the subacromial space and are responsible for the bursal side compression of RC tendons. Intrinsic factors consist of variations in biological, morphological, vascularity, and mechanical properties that are responsible for degenerating RC tendons with shear overload [6]. RC disease has been identified through comorbidities and risk factors such as lateral epicondylitis, use of oral corticosteroids, and diabetes [7]. The main contributing and causative mechanism is excessive mechanical loading [8].

Three varieties of calcific deposits were found with ultrasonography: (1) hyperechoic focus with clear shadow (79%), (2) hyperechoic with dull shadow (14%), and (3) hyperechoic without shadow (7%). Ultrasound (US) is found to be more suitable for large bursal calcifications, but the small scattered deposits were better seen with plain radiography. For detecting and localizing RC calcifications, the US imaging has been proved to be reliable but it could not classify it into formative or resorptive phase [9].

Classification of calcific tendinitis by Mole et al. (French Society of Arthroscopy): type A has dense, homogenous, sharp contours; type B has dense, segmented, sharp contours; type C has heterogeneous, soft contours; and type D shows dystrophic calcifications at the insertion of RC tendons [10].

Various imaging tools like the musculoskeletal US and magnetic resonance imaging (MRI) can help in diagnosing RC disorders [11]. Ultrasonography has been considered an effective imaging modality in the evaluation of both RC and non-RC disorders [12].

The maneuvers used for RC tendinopathy include conservative treatment [13] and physical exercises which are considered as a gold standard treatment option [14]. Other modalities used are acupuncture, massage therapy [15], electrotherapeutic agents [16], NSAIDs, and corticosteroid injections [17] which have also been suggested as treatment options. However, limited evidence is available related to these interventions. Eccentric loading training showed better outcomes in aiding pain and improving functional impairments in patients with RC tendinopathy [18].

Extracorporeal shock wave therapy (ESWT) is considered an effective treatment for treating calcific RC tendinopathy, as it can elicit an analgesic and anti-inflammatory response and promote tissue regeneration [19]. These high-pressure acoustic waves also cause a reduction in enthesopathic pain and enhancement of the functionality of limbs. ESWT is an alternative therapy for calcified and noncalcified RC tendinopathy that can reduce the need for operative treatment.

ESWT has been extensively used for the management of MSK disorders like unhealed fractures, plantar fasciitis, lateral epicondylitis, calcific RC tendinopathies, and heel pain [20]. These multiple mechanisms are involved in the therapeutic effects of ESWT; mechanical stimulation increased regional blood flow and enhanced expression of growth factors [21]. Clinically, two varieties of SWT have been used widely: radial and focused shockwave therapy. Radial shockwaves are generated by accelerating a projectile, through compressed air in a tube, at the end of which it hits an applicator that makes contact with the skin while the focused shockwaves are generated inside the applicator and then focused by a lens and transmitted into the tissue [22].

A systematic review concluded that some studies proved that significant improvement in pain and functionality was observed in subjects with RC tendinopathy by ESWT as compared to conventional treatment. But there is no consensus between these interventions [23].

RC tendinopathy is considered being a highly prevalent medical condition, and physical rehabilitation plays a vital role to recover such patients. However, some studies support its beneficial effects but did not show significant results. Most of the studies compared the effects of shockwave therapy with other modalities; comparison of high-energy SWT was not done with a proper control group to evaluate its effects. Experimental studies were available for showing the effects of shockwaves on plantar fasciitis or lateral epicondylitis, but not much evidence is available for RC tendinopathy. Literature showed a lack of high-quality controlled trials, and some studies had methodological deficiencies; they only emphasize pain and functional outcomes that is why the current trial is designed to determine the effects of high-energy ESWT among patients with shoulder tendinopathy especially on clinical outcomes such as pain intensity, functional disability, and quality of life and also the structural outcomes by checking the US changes.

## 2. Methods

### 2.1. Ethical Approval

This prospective parallel, randomized controlled trial was approved by the Institutional Review Board Committee of the University of Lahore (ref: IRB-UOL-FAHS/693/2020), and it was registered at the Iranian Registry of Clinical Trials (IRCT) (ref: IRCT20200204046373N1 https://irct.ir/trial/45657). The first participant was recruited in the current trial on 28/03/2020. Written informed consent was taken from every participant. Ethical considerations were taken into account, and before treatment, the therapist provided the patients with a form stating that minor bruising, pain, or hematoma is expected in a small number of participants. This trial was conducted at the Physiotherapy Department of Lifeline Health Care and Pain Centre, Lahore Pakistan.

### 2.2. Study Design and Population

From April 2020 to June 2021, participants prediagnosed with calcific RC tendinopathies were asked to participate in the trial. Participants who fulfilled the following selection criteria and presented with similar baseline characteristics were considered eligible: (1) between 30 and 65 years old, (2) painful lateral aspect of shoulder and pain exacerbation with overhead activity, (3) both gender, (4) reduced range of motion (ROM), (5) symptoms present from last three months, (6) positive Neer's impingement test, and (7) the US showing calcific changes (types A and B, >10 mm) in the rotator cuff [10]. Patients were excluded if they have (1) marked atrophy or weakness of any shoulder girdle muscle, (2) previous surgery, (4) recent corticosteroid use or nerve blockage, (5) malignancy, and (6) coagulation abnormalities.

Recruited through consecutive sampling, 30 participants were estimated (15 in each group) having 95% confidence interval and 90% power of the study. The 42 participants were randomized into two groups, having 21 patients in each group by adding 20% dropouts. The sample size is calculated using the following formula: *n* = (*Z*_1−*β*_ + *Z*_1−*α*/2_)^2^ + (§_1_^2^ + §_2_^2^)/(*μ*_1_ − *μ*_2_)^2^. Based on evidence [24], a standard deviation of 2 points of the numeric pain rating scale (NPRS) in the experimental group (ESWT+RPT) and 1.5 points in the routine physiotherapy (RPT) group at 12 weeks were considered. A difference in the NPRS level of 1.5 points was considered clinically relevant.

Subjects were randomly allocated to both groups using a computer-generated randomized method, and it had been ensured by the assessor. The randomization allocation was sealed in opaque envelopes. The assessor and biostatistician were blinded about the intervention given until the completion of the trial. The physiotherapist provided the treatment and was not blinded to the study.

### 2.3. Extracorporeal Shockwave Therapy

The experimental group received high-energy extracorporeal shockwave therapy in addition to routine physical therapy (ESWT+RPT). About 2000 shockwaves of 0.32 mJ/mm^2^ per treatment were given as 12 sessions for the first six weeks (two sessions/week) by one physiotherapist only. Routine treatment was administered to the patients in both experimental and routine physiotherapy (RPT) groups in 12 sessions for six weeks (two sessions/week), and it included these items: pulsed short wave diathermy (SWD) with frequency 27.12 MHz [25], ultrasonic therapy (US) with frequency 1.0 MHz and intensity 1.45 w/cm, and transcutaneous electrical nerve stimulator (TENS) 2–200 Hz with output current < 20 Ma with 200 *μ* seconds along with continuous mode. Exercises were comprised of shoulder strengthening and stretching exercises that were performed for 5 s with 10 repetitions for both experimental and RPT groups as shown in Table 1 [26]. Each exercise was performed with ten repetitions, twice a week in 12 sessions [27].

ESWT was given using a radial SWT BLT-6000 device (UK) twice a week for six weeks. Each ESWT session was given for 15-20 minutes in which patients have been treated by 2000 shocks with 120 Hz. The procedure could be slightly painful in the first session, so a low-energy density of 0.03 mJ/mm^2^ was given for the first five minutes and then progressively increasing to 0.32 mJ/mm^2^. ESWT application was performed on all the RC tendons such as teres minor, subscapularis, supraspinatus, and infraspinatus. In successive treatments, an energy density of 0.32 mJ/mm^2^ was used. An isotonic gel was applied before the probe was placed to the patient's shoulder to localize the waves to the target area, and no local anesthetic was given during the procedure. Therapy was performed by positioning the shoulder at the medial and lateral rotations at 15 degrees with the patient placed in an upright sitting posture. In both groups, treatment was given for 45 minutes in each session.

### 2.4. Outcome Measures and Posttreatment Follow-Up

All the participants were evaluated for baseline characteristics such as age, height, weight, and body mass index (BMI) pre- and posttreatment. The pain level was assessed by NPRS and functional disability level with the Constant and Murley scale (CMS) [28]. For the assessment of the quality of life, the Western Ontario rotator cuff index (WORC) was used [29].

**Figure 1:**
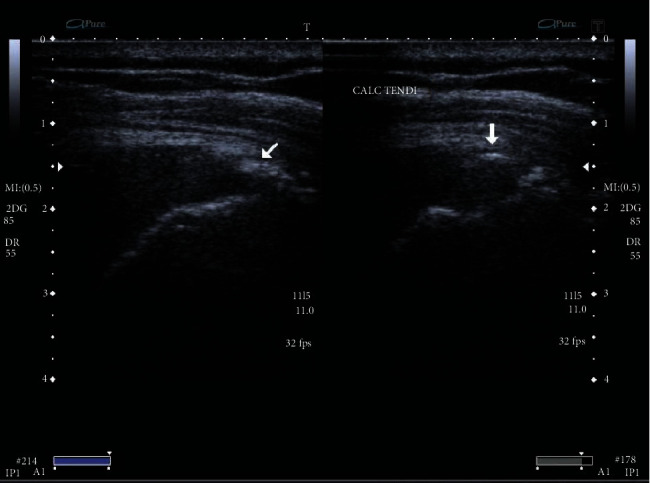
Ultrasonographic changes in calcified supraspinatus tendon (ESWT-RPT group).

**Figure 2:**
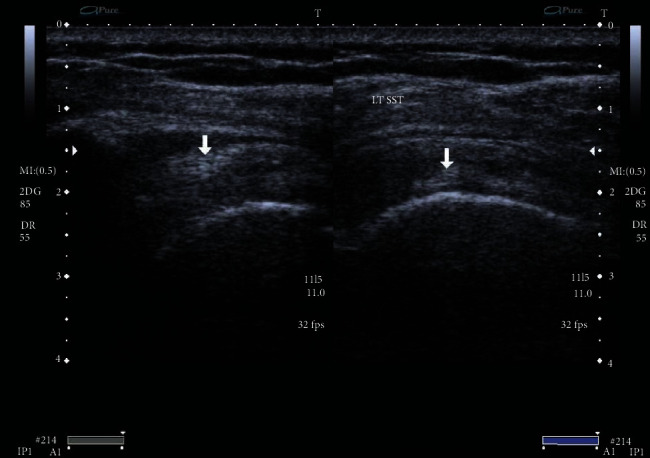
Ultrasonographic changes in calcified supraspinatus tendon (RPT group).

The primary endpoint was the change of the NPRS from baseline to 12 weeks. The 11-point NPRS (0 = no pain, 10 = maximum pain) has been recommended by the Initiative on Methods, Measurement, and Pain Assessment in Clinical Trials as a primary endpoint for chronic pain studies. Secondary endpoints were changes of NPRS at all other follow-up points and changes of the mean CMS and WORC at baseline and 6 and 12 weeks. The CMS has been extensively validated and shows good inter- and intraobserver reproducibility. Ultrasonography was examined to check the size of calcium deposits in pre- and posttreatment sessions in both groups (Figures 1 and 2). The US was based on a standardized protocol; US examinations were performed for the diagnosis of RC pathologies pre- and posttreatment. Changes between pre- and posttreatment radiographs were graded as no, partial (minor changes in mass or appearance), and total or subtotal resorption (80% reduction in calcific masses). Images were evaluated by the musculoskeletal radiologist.

Reliability of musculoskeletal ultrasound imaging to measure calcific deposits in patients with calcified rotator cuff tendinopathy had been done for this study. Validity, reliability, and translation of the WORC had also been done for the current trial.

### 2.5. Statistical Analysis

Using SPSS version 26.0, data analyses were performed. The categorical variables were presented as counts while the continuous variables were presented as means and standard deviations. Normality of data was assessed by the Shapiro-Wilk test, and after fulfilling nonparametric assumptions, the Mann–Whitney *U* test was used to compare both groups at baseline and 6-week and 12-week follow-ups, and the Friedman test was calculated to analyze within-group differences in the mean of each variable between the assessments points (at baseline and 6^th^ week and 12^th^ week after commencement of treatment). The *p* value ≤ 0.05 was considered statistically significant.

## 3. Results

**Figure 3:**
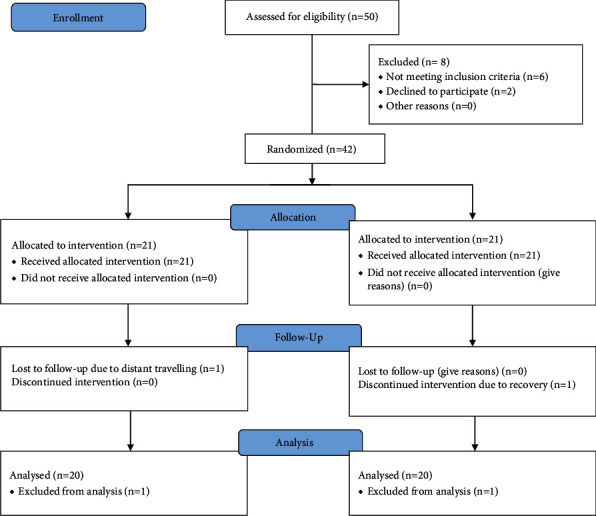
CONSORT flowchart.

In this current trial, 50 subjects were assessed for eligibility but only 42 were selected fulfilling the mentioned criteria. These participants were randomly divided into two groups, having 21 patients. Two participants discontinued the trial at the follow-up stage (*n* = 40) as shown in the CONSORT guidelines (Figure 3), so only 40 patients completed the trial and were analyzed for the results. In the ESWT+RPT and RPT groups, baseline characteristics, i.e., the mean values of age, height, weight, and body mass index, of patients were given (Table 2).

Results showed a significant reduction in the numeric pain rating score and the size of calcific deposits and an improvement in Constant and Murley scores and the quality of life in both groups. There were statistically significant differences regarding NPRS and CMS between the two groups at baseline and 6 and 12 weeks after intervention (*p* < 0.05), shown in Table 3. The pain and functional outcomes showed significant improvement within groups at follow-ups after treatment; their *p* values were found significant as <0.001 (Table 4). Significant results were found in the WORC and ultrasonographic results pre- and posttreatment; more significant results were found in the ESWT group as compared to the RPT group. The effect size at different follow-ups showed a small effect as all the values are less than 0.2 (Table 5).

## 4. Discussion

Calcific tendinopathy is the most dynamic cause of shoulder pain as it is characterized by the presence of calcific deposits in RC tendons [4]. Therefore, the current trial is designed to find the role of ESWT therapy in participants with calcified RC tendinopathy as it is a highly prevalent condition.

In this trial, 50 subjects were assessed for eligibility while 42 were recruited. These subjects were randomly allocated into two groups with 21 patients each. Only 40 patients completed the study and were analyzed for the result findings. Result findings showed a significant reduction in pain intensity, size of calcific deposits, functional activity, and quality of life in both groups. There were statistically significant differences regarding NPRS and CMS at baseline and 6^th^ and 12^th^ weeks and also in WORC and ultrasonographic findings pre- and posttreatment. The effect size at different follow-ups showed a small effect as all the values are less than 0.2.

Louwerens et al. demonstrated that women aged between 30 and 60 years with SAPS and a calcific deposit of >1.5 cm in length had the highest chance of suffering from symptomatic calcific tendinopathy of the RC [4].

In a trial conducted by Duymaz and Sindel in 2019, about 80 patients with calcific RC tendinopathy were randomly recruited into the rESWT group treated with rESWT and conventional PT, and the control group was treated with conventional PT intervention only. All patients were evaluated using the Visual Analog Scale (VAS) and functional disability status with the Disabilities of the Arm, Shoulder, and Hand questionnaire (QuickDASH) before and after treatment. Subjects in the experimental group showed improvement in all scores (all *p* < 0.001) [30]. Effects were assessed with pain intensity, ROM, and functionality, but the effectiveness of treatment was not evaluated through diagnostic imaging techniques. This current trial evaluates the effects using NPRS and CMS and also found the quality of life of these patients. The present study also examines changes in calcific masses ultrasonographically which was incorporated to check the location and amount of calcification before and after treatment.

Albert et al. aimed to compare focused versus radial shockwave therapy for noncalcific RC tendinopathy, with 46 patients randomly divided into 2 groups. The NPRS, CMS, and US findings were the outcome measure tools. Both focused and radial SWT proved to be effective for such patients; however, focused SWT is significantly more effective as compared to radial SWT. The calcific deposit remains unchanged in size in the majority of patients [31]. However, this trial involved calcified RC tendinopathy and used only radial SWT to check the disintegrating effect of shockwaves on calcified deposits.

de Menezes et al. conducted a study to determine the effects of shockwave therapy to a progressive exercise program on shoulder pain and function in patients with RC tendinopathy. The NPRS, CMS, Global Perceived Effects Scale, and Shoulder Pain and Disability Index after three months of follow-up were determined [32].

Klüter et al. had compared the effects of electromagnetic transduction therapy (EMTT) along with ESWT in a study on 86 participants with RC tendinopathy. Both treatment groups experienced significant pain reduction and functional improvement. EMTT combined with shockwave therapy showed significant improvement in pain intensity and functional ability as compared to sham EMTT with ESWT. This study also evaluates effects on pain and function, showing the comparison of EMTT with ESWT [33]. However, this trial proved the effectiveness of ESWT with the control group, and quality of life had been assessed and the size of calcific deposits had been measured using reliable ultrasound.

Another study concluded that ESWT significantly reduces pain and improves functional mobility and quality of life on 384 patients, suffering from different tendinopathies like elbow tendinopathy, Achilles tendinopathy, plantar fasciitis, and RC tendinopathy [34].

A study compared the ESWT group with the transcutaneous electrical nerve stimulation (TENS) group, and US demonstrated that the change in calcific deposit was more in the ESWT group [35].

Li et al., in an experimental study, compared ESWT with the placebo treatment while recruiting 84 patients with RC tendinopathy. Outcome measures were NPRS, CMS, and simple shoulder test score, recorded at 4^th^ and 8^th^ weeks, showing significant results in the ESWT group [36]. Subjective measures were being evaluated in this study, and it did not check the size of calcific deposits; however, objective measures were being evaluated and the quality of life of these patients was being assessed in this current study.

A prospective trial was performed to compare effects of ESWT for RC tendinopathy; 40 patients were recruited, who received 6000 high-energy impulses in three sessions under local anesthesia and low-energy shockwaves. Pain score reduction and functional improvement in CMS were observed, but there is no statistically significant difference found among the groups, after 12-week and one-year follow-up [37]. Significant results were found in this present trial. Because of certain constraints, it is not possible to have a larger sample and follow them for a long duration, but for achieving better effects of this regimen, future exploration should involve well-planned RCTs with extended treatment timeframe, longer follow-up duration, larger sample size, and self-reported measures of function.

A systematic review comprised of 11 nonrandomized trials that involved both calcified and noncalcified tendinopathy. There is moderate evidence about the effectiveness of high-energy ESWT in treating chronic calcified tendinopathy when the SWT is focused on the calcified deposit [38]. The current study provides significant results as the size of calcific deposits gets reduced after applying shockwaves along with routine physical therapy.

More cartilage-specific gene expression and tendon-specific gene downregulation have been reported in studies related to overuse injuries of ST. Because of overuse, some changes take place in which the phenotype of this tendon appears to be cartilage-like [39]. The increased expression of osteopontin and tTG2 could thus be compatible with their enhanced production in the calcific area, probably by osteoclast-like cells involved in the resorptive phase [40]. More studies should be done which can evaluate changes at a molecular or genetic level; this is lacking in this study.

The limitations of the study were that the participants were not observed beyond 12 weeks because of time constraints. Detailed and repeated measurements were not made due to the short time and more outcome measures. The number of participants who participated in the study is low to generalize the outcomes for the whole population. Further investigation is required to check the extent to which our findings can be generalized.

Fewer quantitative studies were available on finding the effects of ESWT. Therefore, this current trial was done to prove its remarkable effects on clinical outcomes in patients with RC tendinopathy. Experimental studies were available for showing the effects of shockwaves on plantar fasciitis or lateral epicondylitis, but not much evidence is available for shoulder tendinopathy. Significant differences were found between pre- and posttreatment scores of pain, functional and structural outcomes within and between the groups, so the alternate hypothesis is considered true.

## 5. Conclusion

High-energy shockwave therapy has been proved to be effective for the management of calcified rotator cuff tendinopathy, improving the pain, functionality, and quality of life of these participants and decreasing the size of calcified deposits. Patients can improve their activities of daily life and reduce their disability with this noninvasive, cost-effective, and safe intervention. Both the groups are effective in patients with RC tendinopathy, but the shockwave therapy is proved to be superior to routine physiotherapy at this long-term follow-up.

## Figures and Tables

**Table 1:** Interventional groups.

ESWT+RPT group	RPT group
(I) Stretching exercises Shoulder external rotation stretch Cross-body posterior stretch Stretch for anterior aspect of the shoulder Shoulder flexion stretch (II) Strengthening exercises Chair press Restricted scapular retraction Restricted scapular protraction Shoulder abduction “scaption” (0°-90°) with theraband Shoulder scapular extension with theraband (III) Shockwave therapy	(I) Stretching exercises Shoulder external rotation stretch Cross-body posterior stretch Stretch for anterior aspect of the shoulder Shoulder flexion stretch (II) Strengthening exercises Chair press Restricted scapular retraction Restricted scapular protraction Shoulder abduction “scaption” (0°-90°) with theraband Shoulder scapular extension with theraband

ESWT±RPT: extracorporeal shockwave therapy and routine physiotherapy group; RPT: routine physiotherapy group.

**Table 2:** Baseline characteristics.

	ESWT±RPT group (mean ± S.D.)	RPT group (mean ± S.D.)
Age (years)	48.7 ± 6.74	49.8 ± 7.54
Height (m)	1.68 ± 0.09	1.67 ± 0.06
Weight (kg)	76.10 ± 12.17	77.85 ± 10.86
BMI	27.10 ± 53.94	27.99 ± 3.50

ESWT±PT: extracorporeal shockwave therapy and routine physiotherapy; RPT: routine physiotherapy; S.D.: standard deviation; m: meters; kg: kilogram; BMI: body mass index.

**Table 3:** Between-group differences for NPRS, CMS, US, and WORC.

	Groups	*N*	Mean	Standard deviation	Mean rank	*p* value
NPRS (baseline)	ESWT+RPT group	20	7.8	1.36	19.75	0.677
	RPT group	20	7.9	1.20	21.25	
NPRS (6^th^ week)	ESWT+RPT group	20	5.0	1.62	16.68	0.035
	RPT group	20	6.0	1.48	24.33	
NPRS (12^th^ week)	ESWT+RPT group	20	3.3	1.78	19.03	0.041
	RPT group	20	4.75	1.94	21.98	
CMS (baseline)	ESWT+RPT group	20	35.8	13.44	21.90	0.448
	RPT group	20	34.3	13.98	19.10	
CMS (6^th^ week)	ESWT+RPT group	20	54.5	11.64	20.85	0.850
	RPT group	20	55.8	16.09	20.15	
CMS (12^th^ week)	ESWT+RPT group	20	75.2	13.35	21.53	0.057
	RPT group	20	72.4	15.45	19.48	
US (pretreatment)	ESWT+RPT group	20	13.8	1.21	18.50	0.082
	RPT group	20	14.4	0.00	22.50	
US (posttreatment)	ESWT+RPT group	20	12.46	2.00	20.30	0.035
	RPT group	20	12.4	1.55	20.70	
WORC (pretreatment)	ESWT+RPT group	20	37.1	14.35	20.93	0.817
	RPT group	20	36.9	13.90	20.08	
WORC (posttreatment)	ESWT+RPT group	20	70.55	15.26	21.25	0.038
	RPT group	20	67.9	16.1	19.75	

NPRS: numeric pain rating scale; CMS: Constant and Murley score; US: ultrasound findings; WORC: Western Ontario rotator cuff index; *p* value: ≤0.05 considered significant; ESWT±RPT: extracorporeal shockwave therapy and routine physiotherapy group; RPT: routine physiotherapy group.

**Table 4:** Within-group differences for NPRS and CMS.

	Mean rank	Chi-square	df	*p* value
NPRS (baseline)	2.91	69.208	2	<0.0001
NPRS (6^th^ week)	2.00			
NPRS (12^th^ week)	1.09			
CMS (baseline)	1.00	80.000	2	<0.0001
CMS (6^th^ week)	2.00			
CMS (12^th^ week)	3.00			

NPRS: numeric pain rating scale; CMS: Constant and Murley score; ESWT±RPT: extracorporeal shockwave therapy and routine physiotherapy group; RPT: routine physiotherapy group. *p* value: ≤0.05 considered significant.

**Table 5:** NPRS, CMS, US, and WORC at different follow-ups.

Outcome variables	Type III sum of squares	df	Mean square	*F*	Sig	Partial eta squared
NPRS baseline	0.100	1	0.100	0.060	0.807	0.002
NPRS 1^st^ follow-up	10.000	1	10.000	4.130	0.049	0.098
NPRS 1^st^ follow-up	2.025	1	2.025	0.583	0.450	0.015
CMS baseline	21.025	1	21.025	0.112	0.740	0.003
CMS 1^st^ follow-up	15.625	1	15.625	0.079	0.780	0.002
CMS 2^nd^ follow-up	75.625	1	75.625	0.362	0.551	0.009
US pretreatment	3.025	1	3.025	4.107	0.050	0.098
US posttreatment	0.004	1	0.004	0.001	0.972	0.000
WORC pretreatment	0.625	1	0.625	0.003	0.956	0.000
WORC posttreatment	70.225	1	70.225	0.284	0.597	0.007

NPRS: numeric pain rating scale; CMS: Constant and Murley score; US: ultrasound findings; WORC: Western Ontario rotator cuff index. *p* value: ≤0.05 considered significant.

## Data Availability

Data will be available on request. The corresponding author will submit all dataset files.

## Supplementary Materials

Supplementary Materials CONSORT guidelines. All methods were performed following the relevant guidelines and regulations.
