# Supplementary material for: Effects of High-Energy Extracorporeal Shockwave Therapy on Pain, Functional Disability, Quality of Life, and Ultrasonographic Changes in Patients with Calcified Rotator Cuff Tendinopathy
Source: Biomed Res Int. 2022 Mar 4;2022:1230857. doi: 10.1155/2022/1230857 (PMC8916860; doi:10.1155/2022/1230857)
Supplement: Supplementary Materials — CONSORT guidelines. All methods were performed following the relevant guidelines and regulations. [file 1230857.f1.zip › SUPPLEMENTARY DESCRIPTION_1230857.docx]

SUPPLEMENTARY DESCRIPTION:

CONSORT 2010 checklist of information to include when reporting a randomised trial
